# Ultra high throughput sequencing excludes *MDH1* as candidate gene for *RP28*-linked retinitis pigmentosa

**Published:** 2009-12-08

**Authors:** Thomas Rio Frio, Sylwia Panek, Christian Iseli, Silvio Alessandro Di Gioia, Arun Kumar, Andreas Gal, Carlo Rivolta

**Affiliations:** 1 Department of Medical Genetics, University of Lausanne, Lausanne, Switzerland; 2 Ludwig Institute for Cancer Research and Swiss Institute of Bioinformatics, Lausanne, Switzerland; 3 Department of Molecular Reproduction, Development and Genetics, Indian Institute of Science, Bangalore, India; 4 Institut für Humangenetik, Universitätsklinikum Hamburg-Eppendorf, Hamburg, Germany

## Abstract

**Purpose:**

Mutations in IDH3B, an enzyme participating in the Krebs cycle, have recently been found to cause autosomal recessive retinitis pigmentosa (arRP). The *MDH1* gene maps within the *RP28* arRP linkage interval and encodes cytoplasmic malate dehydrogenase, an enzyme functionally related to IDH3B. As a proof of concept for candidate gene screening to be routinely performed by ultra high throughput sequencing (UHTs), we analyzed *MDH1* in a patient from each of the two families described so far to show linkage between arRP and *RP28*.

**Methods:**

With genomic long-range PCR, we amplified all introns and exons of the *MDH1* gene (23.4 kb). PCR products were then sequenced by short-read UHTs with no further processing. Computer-based mapping of the reads and mutation detection were performed by three independent software packages.

**Results:**

Despite the intrinsic complexity of human genome sequences, reads were easily mapped and analyzed, and all algorithms used provided the same results. The two patients were homozygous for all DNA variants identified in the region, which confirms previous linkage and homozygosity mapping results, but had different haplotypes, indicating genetic or allelic heterogeneity. None of the DNA changes detected could be associated with the disease.

**Conclusions:**

The *MDH1* gene is not the cause of *RP28*-linked arRP. Our experimental strategy shows that long-range genomic PCR followed by UHTs provides an excellent system to perform a thorough screening of candidate genes for hereditary retinal degeneration.

## Introduction

Retinitis pigmentosa (RP; OMIM 268000) is a hereditary and progressive form of retinal degeneration, with an estimated prevalence of one patient in 4,000 people [1]. Affected individuals experience the constant and unstoppable death of photoreceptors, a phenomenon that results in increasing loss of sight and in many instances legal or complete blindness [2]. Genetically, RP is a highly heterogeneous condition, since around 50 genes or loci, most of which act as individual Mendelian entities, have been implicated so far (RetNet).

**Figure 1:**
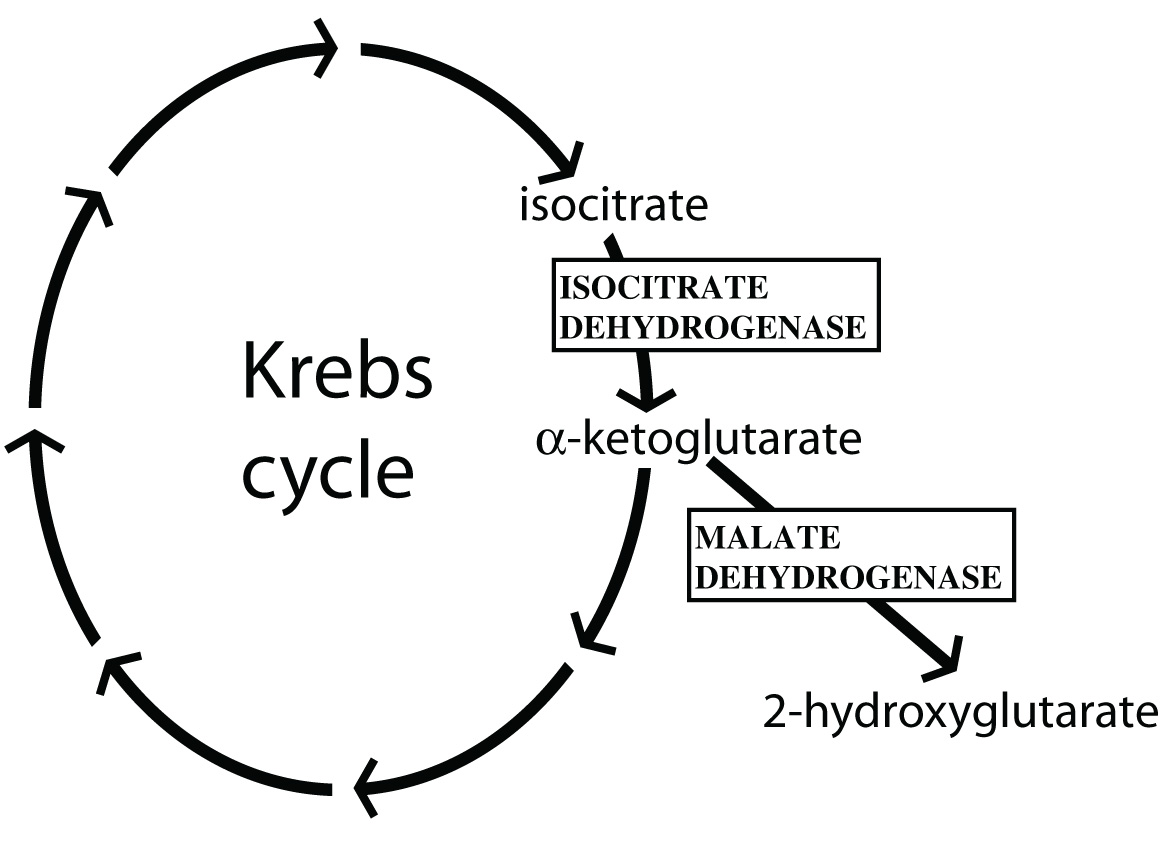
Schematic representation of the Krebs cycle. The specific functions of two enzymes, isocitrate dehydrogenase (NAD+ dependent, partly encoded by *IDH3*) and the *MDH1*-encoded malate dehydrogenase, are shown.

The *RP28* locus associated with autosomal recessive RP (arRP) has previously been mapped to chromosome 2p14-p15 through the analyses of two consanguineous but apparently unrelated Indian families [3,4]. The candidate region spans 1.06 cM and includes 15 genes, 14 of which are expressed in the retina. None of these genes has been previously associated with retinal degeneration or is known to have a specific function in the retina. The *IDH3B* gene, which encodes for the β subunit of isocitrate dehydrogenase 3 (NAD+ dependent, EC 1.1.1.41), was recently found to be associated with arRP, indicating a link between the Krebs cycle and retinal disease [5]. One of the genes within the *RP28* linkage interval, *MDH1*, encodes for the cytosolic form of malate dehydrogenase (EC 1.1.1.37), which is directly connected to the Krebs cycle via the malate-aspartate shuttle. The gene products of *IDH3B* and *MDH1* are related at an additional functional level, since malate dehydrogenase can convert the product of isocitrate dehydrogenase [6] (Figure 1). Therefore, we reasoned that *MDH1* could correspond to the *RP28* locus, and mutations in its sequence could be responsible for the disease in a manner similar to that of pathogenic changes in *IDH3B*.

A common approach to the mutational screening of candidate genes consists of sequencing their exons and immediate intron boundaries. However, since pathogenic mutations can sometimes be located deep within introns, as was recently shown for retinal degeneration genes as well [7,8], we decided to analyze the full *MDH1* sequence. To circumvent problems linked to sequence length and composition and to test the potential of parallel sequencing in routine mutation detection, we used long-range PCR (LR-PCR) amplification of genomic DNA followed by ultra high throughput sequencing (UHTs).

## Methods

### DNA samples and long-range PCR

The genomic DNA used in this study was part of the collection of samples that originally allowed mapping of the *RP28* locus. Samples were obtained in accordance with the ethical guidelines regulating these previous investigations [3,4]. Specifically, they belonged to patient V-4 from family PMK146 [3] and patient IV-7 from family IIS-2 [4]. The entire *MDH1* gene (nine exons and eight introns) as well as an additional 0.5 kb upstream and 4.7 kb downstream (23,459 bp in total, from nucleotide 63,669,094 to nucleotide 63,692,552 of chromosome 2, NCBI *Homo sapiens* Build 36.3, NC_000002.10) were amplified by three tiled LR-PCRs, using the primer pairs described in Table 1. PCR reaction mixes were set as specified by the standard protocol of TaKaRa LA Taq (TaKaRa Bio Inc., Shiga, Japan) using GC Buffer I, except the final reaction volume was reduced to 10 µl, and the concentration of each primer to 0.1 µM. The thermal profile common to all sets of primers consisted of an initial step at 94 °C for 1 min, followed by 30 cycles at 98 °C for 5 s and 68 °C for 15 min, and a final step at 72 °C for 10 min. Prior to sequencing, LR-PCR products were checked on agarose gel, quantified using the ImageJ software [9], and pooled in roughly equimolar quantities.

**Table 1:** Primer pairs used in LR-PCRs.

**Nucleotide sequence (5′-3′)**	**Size of PCR product (bp)**
F: TGTCCGGTCGTCCCAACTTATCAATTC	11,429
R: CTGGTCACTGGCTCCTTGGCATACTTATCTAT	
F: CAAGGAGAACTTCAGTTGCTTGACTCGTTT	6,752
R: AACACCATAGGAGTTGCCATCAGAGATAACAC		
F: TGAGGATTAGGTTTCCCTGGCCTACTTCAC	6,204	
R: TCAATTGTGCTACCCAGGTCAGGCTATGA		

Abbreviations: F represents forward primer; R represents reverse primer.

### Ultra high throughput (UHT) and conventional Sanger sequencing

UHTs was performed with an Illumina Genome Analyzer (Illumina, San Diego, CA) for the DNA of patient IV-7/IIS-2 and an Illumina Genome Analyzer II (Illumina) with the paired-end procedure for the DNA of patient V-4/PMK146, according to the manufacturer’s protocols and starting from around 400 ng of LR-PCR products.

Conventional Sanger sequencing was performed using exon-specific primers (Table 2) and the BigDye Terminator v1.1 cycle sequencing kit (Applied Biosystems, Foster City, CA) on LR-PCR products purified by treatment with ExoSAP-IT (USB, Cleveland, OH). Sequencing reactions were purified on Performa DTR columns (Edge BioSystems, Gaithersburg, MD) and run on an ABI-3130XL (Applied Biosystems).

**Table 2:** Primers used in Sanger sequencing.

**Nucleotide sequence (5′-3′)**	**Exon**
GCTCATCCTCAGGGACTACTTTGCAATC	1
CCAGGGTTTGGATCACCACATACTGAAC	2
GTGGTGATTCCTCTCACTGTGTCTGTTAGC	3
CTCAGGCTCCTGAAATGTATATCAGTGTG	4
ATCAAGTAGGAAGTCCAGCCTCT	5
GTCCACAGTTGTTACCACTGTTAAGCTG	6
GCCAGTCATGATCTAGTGTGATCTGATGTG	7
CCTGGTGCTGATGATAGTTCCTTACACA	8
GTAGAGATGGGGTGTCACTATTTG	9

### Sequence analysis

Mapping and analysis of UHTs reads were performed independently with the use of three different software packages: FetchGWI/align0 [10,11], Maq [12], and the CLC Genomics Workbench (CLC bio, Aarhus, Denmark). In all instances, analyses were performed on the original calls generated by the Genome Analyzer. The procedure followed when using the FetchGWI/align0 packages was composed of multiple sequential steps. First, all the reads were filtered according to the Phred quality score of each nucleotide, replacing nucleotides with low scores by “Ns” and discarding reads with more than three Ns. Second, fetchGWI was used to find unique exact matches of all the retained reads. Reads that mapped within the target regions were directly used, while those mapping outside of these regions were kept as a negative control group. Third, among reads for which it was impossible to find any matches (either unique or repeated), those sharing a common 12-mer (except mono- or di-nucleotide repeats) with the selected regions were kept and aligned against them by a global Smith-Waterman procedure (by align0). Fourth, reads that had a good score (no more than three mismatches) were verified not to produce a better alignment score against the negative control group kept during the first step. Finally, DNA variants were derived from sequences retained at step #1 and from the global alignment outputs produced at step #3. Mapping of sequences and mutation detection via Maq were accomplished as follows. First, Maq performed ungapped alignments of the reads on the reference sequence by retaining only reads with one or no mismatches in the first 24 bases and a maximum of one mismatch for the remaining bases. After mapping, Maq generated a consensus sequence and calculated the Phred quality score at each position of this sequence. Results from the analysis allowed the calling of genomic variations compared to reference sequence. The requirements for calling a variation were a minimum Phred quality score of 40; no insertions or deletions in the range of five bases; only an additional single nucleotide polymorphism (SNP) within a ten-base window; a minimum neighbor Phred quality score of 20; and a minimum coverage of 3×. The CLC Genomics Workbench-assisted mapping and mutation detection were conducted according to the general procedure recommended by the software house. More specifically, the raw sequences first underwent a filtering and trimming process based on the quality of their scores (quality score cutoff=0.01, maximum number of ambiguous nucleotides=2). All reads satisfying these criteria were then mapped on the 23,459-bp reference sequence mentioned above using the Reference Assembly algorithm within the UHTs module of the CLC package (mismatch cost=2, indel costs=3). Detection of variants was performed by identifying on the final mapping all bases for which 60% or more of the calls differed from the reference sequence, regardless of the base coverage.

Electropherograms generated by Sanger sequencing were analyzed either by the Staden software package [13] or the CLC Genomics Workbench (CLC bio).

## Results

We sequenced 23,459 bp of human chromosome 2, which span the *MDH1* gene, in patients V-4/PMK146 and IV-7/IIS-2 by UHTs. All three software packages used to filter and align the reads provided essentially the same results and identified the same DNA variants, all of which were intronic (Table 3). As expected, affected members from the two consanguineous families displayed homozygosity for all DNA changes present in the region. This supports the hypothesis that two copies of the same recessive mutation were inherited by patients from a single ancestor who was common to both their paternal and maternal branches, as previous microsatellite analyses have suggested [3,4]. Since all of the identified variants represented either known SNPs or changes with no predicted effects on splicing [14], none of them was considered potentially pathogenic. Sanger sequencing of all *MDH1* exons and their intron vicinities confirmed the absence of DNA variants with respect to the reference sequence, apart from seven SNPs already detected by UHTs: rs10469944, rs2305157, rs7606045, rs2604613, rs262472, rs262473 (in V-4/PMK146) and g.42648177T>A (in IV-7/IIS-2).

**Table 3:** Variants detected in patients V-4/PMK146 and IV7/IIS-2.

**Patient**	**Variant #**	**dbSNP entry**	**HGVS name (position)**
V-4/PMK146	1	rs10469944	NT_022184.14:g.42632605C>G
	2	rs10469945	NT_022184.14:g.42632709T>C
	3	rs6546018	NT_022184.14:g.42633355G>T
	4	rs6546019	NT_022184.14:g.42633373A>G
	5	rs1446569	NT_022184.14:g.42634247C>G
	6	rs4671519	NT_022184.14:g.42636331C>T
	7	rs2305157	NT_022184.14:g.42638718T>C
	8	rs10865340	NT_022184.14:g.42639982C>G
	9	rs10865341	NT_022184.14:g.42639983A>T
	10	rs11125979	NT_022184.14:g.42640239T>G
	11	rs4671069	NT_022184.14:g.42641337C>A
	12	rs1255	NT_022184.14:g.42641867T>C
	13	rs7606045	NT_022184.14:g.42642461T>G
	14	rs964880	NT_022184.14:g.42642865C>A
	15	rs2121351	NT_022184.14:g.42644294G>A
	16	rs262470	NT_022184.14:g.42646492C>T
	17	rs262471	NT_022184.14:g.42646802G>A
	18	rs2604613	NT_022184.14:g.42648709G>A
	19	rs262472	NT_022184.14:g.42649549G>A
	20	rs262473	NT_022184.14:g.42649746G>T
	21	rs262474	NT_022184.14:g.42650552A>G
	22	rs262489	NT_022184.14:g.42652200G>A
	23	rs262490	NT_022184.14:g.42653584A>G
IV-7/IIS-2	1	NR	NT_022184.14:g.42645344C>G
	2	NR	NT_022184.14:g.42648177T>A

NR, not referenced in public databases.

The large majority of UHTs reads were of good quality, as only a minimal part of them were discarded by the filtering procedures. Furthermore, filtered sequences could be easily mapped back to the reference sequence of the human genome, with an overall success rate of about 97%. The average length of the reads (30.5 bases) was also satisfying and, given the amount of sequences that were generated, produced a very high mean base coverage. More specifically, sequencing runs produced 5.2 million and 4.8 million raw sequences when the LR-PCRs from patients V-4/PMK146 and IV-7/IIS-2, respectively, were used as a template. Following the Maq procedure, 5.1 million and 4.5 million sequences from these two samples were retained after filtering for quality, generating an average coverage of 7,600× and 6,700× per given base. Slightly lower values were obtained with the CLC bio and FetchGWI/align0 packages. For CLC bio, 4.9 million (V-4/PMK146) and 3.8 million (IV-7/IIS-2) reads from the raw sequences satisfied filtering criteria, producing an average coverage of 7,000× and 4,700×, respectively. For the FetchGWI/align0 procedure, the corresponding values were 5.0 million and 3.1 million sequences, resulting in a mean coverage of 6,500× and 3,700×.

To determine the limits of UHTs as a potential tool for routine mutation detection in a homozygous context, we simulated various coverage values by randomly selecting 500,000, 50,000, 25,000, 10,000, and 5,000 filtered reads from the V-4/PMK146 sample. We then remapped them onto the 23,459-bp reference contig with the CLC bio software, exactly as performed on the original set of sequences. Although the average coverage was substantially different across the sets, as well as proportional to the simulated coverage (Table 4), we did not observe any dramatic reduction in power for the correct detection of SNPs. Specifically, whenever coverage of at least 2× could be preserved, all SNPs were correctly called (Table 4). Below this threshold, false positives began to appear, as expected, since technical sequencing errors could not be verified by any overlapping sequence (data not shown).

**Table 4:** Variants detected in patient V-4/PMK146 as a function of base coverage.

**SNP**	**Reference base**	**Calls in patient V-4/PMK146 (absolute coverage of the base)**					
		**4,000,000** **sequences**	**500,000** **sequences**	**50,000** **sequences**	**25,000** **sequences**	**10,000** **sequences**	**5,000** **sequences**
rs10469944	C	G (3,901)	G (332)	G (34)	G (22)	G (7)	G (2)
rs10469945	T	C (3,602)	C (375)	C (35)	C (22)	C (6)	C (4)
rs6546018	G	T (3,445)	T (315)	T (30)	T (18)	T (8)	T (5)
rs6546019	A	G (3,403)	G (316)	G (24)	G (13)	G (5)	G (4)
rs1446569	C	G (3,407)	G (327)	G (36)	G (9)	G (5)	G (2)
rs4671519	C	T (4,079)	T (375)	T (35)	T (15)	T (6)	T (4)
rs2305157	T	C (2,671)	C (267)	C (28)	C (11)	No Call	No Call
rs10865340	C	G (2,227)	G (221)	G (27)	G (11)	G (3)	G (2)
rs10865341	A	T (2,228)	T (221)	T (27)	T (11)	T (3)	T (2)
rs11125979	T	G (1,899)	G (172)	G (10)	G (9)	G (2)	No Call
rs4671069	C	A (3,053)	A (275)	A (27)	A (14)	A (7)	No Call
rs1255	T	C (2,588)	C (249)	C (25)	C (15)	C (5)	No Call
rs7606045	T	G (2,390)	G (227)	G (19)	G (13)	G (8)	G (2)
rs964880	C	A (5,158)	A (480)	A (52)	A (24)	A (12)	A (7)
rs2121351	G	A (6,998)	A (684)	A (51)	A (29)	A (14)	A (7)
rs262470	C	T (6,461)	T (620)	T (60)	T (29)	T (13)	T (5)
rs262471	G	A (6,379)	A (545)	A (56)	A (31)	A (13)	A (6)
rs2604613	G	A (5,445)	A (514)	A (69)	A (32)	A (9)	A (4)
rs262472	G	A (7,326)	A (732)	A (70)	A (35)	A (12)	A (6)
rs262473	G	T (3,401)	T (308)	T (33)	T (13)	T (7)	T (3)
rs262474	A	G (7,445)	G (635)	G (51)	G (35)	G (20)	G (5)
rs262489	G	A (6,908)	A (528)	A (47)	A (33)	A (9)	A (6)
rs262490	A	G (4,928)	G (469)	G (39)	G (16)	G (9)	G (2)
W.S. Avg. Cov.		7,053	659	64.5	32.4	12.8	6.3
SNP Avg. Cov.		4,319	399	38.5	20	8.3	4.1

No Call, base call not performed (coverage<2); W.S. Avg. Cov., average base coverage for the whole sequence; SNP Avg. Cov., average base coverage of all detected SNPs.

## Discussion

Identification of RP genes can be performed via genome-wide linkage analyses (when large families with multiple affected individuals are available), candidate gene screenings (when several dozen unrelated patients can be investigated), or a combination of both techniques. Good candidate genes share sequence similarities with other disease genes, relate to the same biochemical pathway, or are expressed mainly or exclusively in the affected tissue or organ [15,16]. To increase the chances of gene identification, the systematic screening of all genes present within an interval defined by linkage mapping has also been adopted, allowing the discovery of mutations in genes that, a priori, were not considered prime candidates [17,18].

Regardless of the strategy used to select the candidate genes, in most cases only exonic and nearby intronic sequences are investigated, for a few practical reasons. Specifically, exons and surrounding splicing signals are more likely to harbor disease-causing mutations than deep intronic regions. In addition, they are shorter and cheaper to analyze as well as easier to sequence, since they rarely contain repeats or low-complexity regions. However, by definition, this strategy prevents the identification of deep intronic mutations.

In this study, we perform a mutational screening on a candidate gene that maps within a previously identified linkage interval by using UHTs to analyze its introns and exons. We exclude *MDH1* as a candidate gene for *RP28*-linked arRP and determine that patients from the only RP28 families identified so far carry different haplotypes in this region. Under the assumption that the disease is caused by an *RP28* mutation in both families, our finding suggests that these Indian pedigrees are truly unrelated. The absence of a common disease haplotype implies the absence of a founder mutation and, furthermore, does not exclude the possibility that two distinct arRP loci could lie in this region. Therefore, the 13 other genes present in the *RP28* interval and expressed in the retina remain to be screened, likely by a global and blind sequencing strategy. Unlike *MDH1*, no biologic feature currently points to another promising candidate. Since several genes have already been shown to cause RP despite their apparent irrelevance to retinal physiology as well as their ubiquitous expression, the hypothesis that a non-obvious candidate could be the *RP28* gene is not particularly surprising.

However, in addition to these specific results, our work can be considered a proof of concept experiment for the use of highly parallel sequencing techniques for mutation detection in monogenic diseases. The assumption that UHTs could replace conventional sequencing in human gene screening procedures is not completely straightforward, since the former technique has a series of defects (e.g., short read length and relatively poor accuracy in base calling) that render it not particularly suitable for the analysis of the human genome [19]. Although we have willingly reduced the complexity of our analyses by selecting homozygous DNA regions in patients from consanguineous families, our results indicate that mutation detection via UHTs could be performed even when coverage is relatively low. Factors such as heterozygozity (e.g., in dominant diseases) or pooling of multiple samples unquestionably increase the noise and decrease the power of UHTs analyses. However, recent studies have shown that, when specific countermeasures are adopted, these confounding elements can be reduced or eliminated [20-22].

In summary, we believe that the strategy used for the analysis of the *MDH1* gene likely represents one of the technical approaches to future candidate gene screening in monogenic conditions. Since retinitis pigmentosa and allied diseases show great genetic heterogeneity, the screening of large sets of patient DNA samples is required. Therefore, UHTs, in combination with systematic long-range PCR [23-25] or genomic sequence capturing [26,27], can be particularly relevant in the analysis of this disorder.
